# Supplementary material for: Autoantibody binding and unique enzyme-substrate intermediate conformation of human transglutaminase 3
Source: Nat Commun. 2023 Oct 5;14:6216. doi: 10.1038/s41467-023-42004-z (PMC10556103; doi:10.1038/s41467-023-42004-z)
Supplement: Supplementary file 1 — Supplementary Information [file 41467_2023_42004_MOESM1_ESM.pdf]

Supplementary Information for

Autoantibody binding and unique enzyme-substrate intermediate conformation of human transglutaminase 3

Julie Elisabeth Heggelund[1,2*], Saykat Das[1,2], Jorunn Stamnaes[1,2], Rasmus Iversen[1,2] and Ludvig Magne Sollid[1,2*]

[1] KG Jebsen Coeliac Disease Research Centre, Institute of Clinical Medicine, University of Oslo, Oslo, Norway, [2] Department of Immunology, Oslo University Hospital-Rikshospitalet, Oslo, Norway.

[*] Corresponding authors: Julie Elisabeth Heggelund, e-mail: j.e.heggelund@medisin.uio.no and Ludvig M. Sollid, e-mail: l.m.sollid@medisin.uio.no

Contents:

Supplementary Figure 1. SDS-PAGE analysis of a crystal of TG3 zymogen+Fab.

Supplementary Figure 2. ESI MS/MS spectra of TG3 with Z-DON.

Supplementary Figure 3. Size exclusion chromatographic analysis of cathepsin L cleaved TG3 with and without Z- DON.

Supplementary Table 1. Overview of selected crystal structures of TG3, TG2 and FXIIIA.

Supplementary Table 2. Data collection and refinement statistics.

Supplementary Table 3. Analysis of metal binding sites using the CheckMyMetal server.

Supplementary Table 4. Overview of hydrogen bonds between Fab DH63-B02 and TG3.

Supplementary Table 5. Overview of hydrogen bonds between Fab DH63-B02 and TG3 C1C2 domains.

Supplementary Table 6. Overview of direct hydrogen bonds between residues in the catalytic and C1C2 domains of TG3.

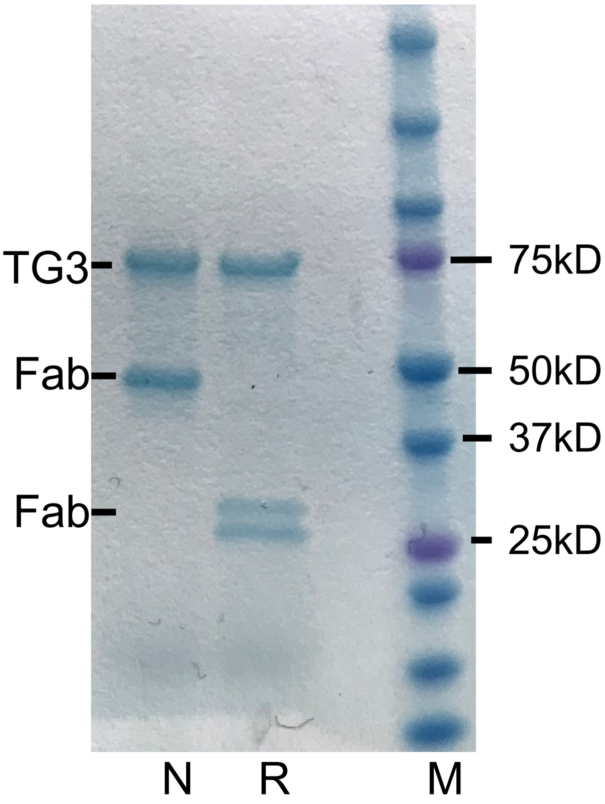

Supplementary Figure 1. **SDS-PAGE analysis of a crystal of TG3 zymogen+Fab.** N. non-reducing conditions showing the intact Fab (47 kDa) and intact TG3 (79 kDa). R. Reducing conditions showing the individual heavy- and light chains of the Fab and the intact TG3. Source data are provided as a Source Data file.

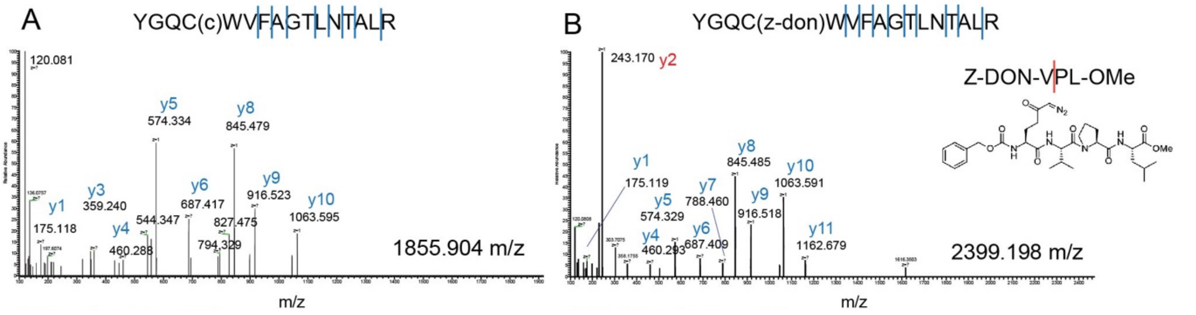

Supplementary Figure 2. **ESI MS/MS spectra of TG3 with Z-DON.** Fragment-ion (y2) from the inhibitor peptide backbone is indicated in red. (c = carbamidomethyl). **a** ESI MS/MS spectra of tryptic peptides harbouring the active site Cys273 from digest of dispase cleaved unmodified TG3. **b** ESI MS/MS spectra of tryptic peptides harbouring the active site Cys273 from digest of dispase cleaved TG3 incubated with Z-DON and $CaCl_2$.

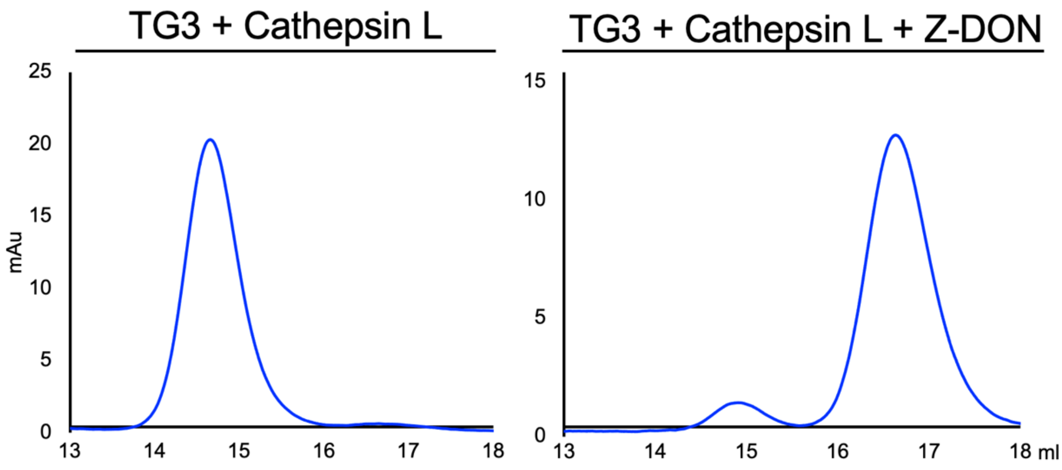

Supplementary Figure 3. **Size exclusion chromatographic analysis of cathepsin L cleaved TG3 with and without Z-DON.** Blue line, absorbance at 280 nm. Source data are provided as a Source Data file.

Supplementary Table 1. Overview of selected crystal structures of TG3, TG2 and FXIIIA

PDB ID	Reference	Resolution	Ca^{2+} sites	State / Ligand	Comment	C1C2
**TG3**						
1NUF	(Ahvazi *et al*, 2003)	2.7Å	$1Ca^{2+}$ site 1	-	Substrate site covered by Asp325 loop and C1, dyad formed	closed
1L9M	(Ahvazi *et al*, 2002)	2.5Å	$1Ca^{2+}$ site 1	zymogen	Substrate site covered by Asp325 loop and C1, dyad formed	closed
1NUG	Ahvazi *et al*, 2003)	2.4Å	$2Ca^{2+}$ site 1, 2 $1Mg^{2+}$ site 3	-	Substrate site covered by Asp325 loop and C1, dyad formed	closed
1NUD	Ahvazi *et al*, 2003)	2.7Å	$3Ca^{2+}$	-	Asp310 not binding in Ca^{2+} site 3, C1 covering substrate site, dyad formed	closed
1L9N	Ahvazi *et al*, 2002)	2.1Å	$3Ca^{2+}$	-	Asp310 not binding in Ca^{2+} site 3, C1 covering substrate site, dyad formed	closed
8OXV	This work	1.8Å	$3Ca^{2+}$	zymogen	Asp310 not binding in Ca^{2+} site 3, C1 covering substrate site, dyad formed	closed
8OXW	This work	1.7Å	$3Ca^{2+}$	-	Asp310 not binding in Ca^{2+} site 3, C1 covering substrate site, dyad formed	closed
8OXX	This work	2.5Å	$3Ca^{2+}$	Z-DON (Z006)	Asp310 binding Ca^{2+} site 3, Trp328 rotated, dyad formed	missing
8OXY	This work	2.0Å	-	-	Substrate site covered by Asp325 loop and C1, dyad not formed.	closed
**TG2**						
1KV3	(Liu *et al*, 2002)	2.8Å	-	GDP	No co-ordination in Ca^{2+} sites, dyad not formed	closed
2Q3Z	(Pinkas *et al*, 2007)	2.0Å	-	Peptide inhibitor	No co-ordination in Ca^{2+} sites, dyad not formed	extended
3S3J	-	2.25Å	-	Z-DON (Z006)	No co-ordination in Ca^{2+} sites, dyad not formed	extended
3S3P	-	2.5Å	-	ZED754 (Z013)	No co-ordination in Ca^{2+} sites, dyad not formed	extended
3S3S	-	2.3Å	-	ZED754 derivative	No co-ordination in Ca^{2+} sites, dyad not formed	extended
3LY6	(Han *et al*, 2010)	3.1Å	-	ATP	No co-ordination in Ca^{2+} sites, dyad not formed	closed
4PYG	(Jang *et al*, 2014)	2.8Å	-	GTP	No co-ordination in Ca^{2+} sites, dyad not formed	closed
**FXIIIA**						
1GGT	(Yee *et al*, 1994)	2.65Å	-	Zymogen, N-term peptide	Substrate site covered by N-peptide and C1, dyad not formed.	closed
1FIE	(Yee *et al*, 1995)	2.5Å	-	N-term peptide	Substrate site covered by N-peptide and C1, dyad not formed.	closed
1GGU	(Fox *et al*, 1999)	2.1Å	$1Ca^{2+}$ site 2	N-term peptide	Substrate site covered by N-peptide and C1, dyad not formed.	closed
4KTY	(Stieler *et al*, 2013)	2.0Å	$3Ca^{2+}$	ZED1301	Asp351 binding Ca^{2+} site 3, Trp370 rotated, dyad formed	back
5MHM	(Stieler *et al*, 2020)	2.1Å	$3Ca^{2+}$	ZED1630	Asp351 binding Ca^{2+} site 3, Trp370 rotated, dyad formed	back
5MHN	(Stieler *et al.*, 2020)	2.5Å	$3Ca^{2+}$	ZED2160	Asp351 binding Ca^{2+} site 3, Trp370 rotated, dyad formed	back
5MHO	(Stieler *et al.*, 2020)	2.9Å	$3Ca^{2+}$	ZED2369	Asp351 binding Ca^{2+} site 3, Trp370 rotated, dyad formed	back

Supplementary Table 2. Data collection and refinement statistics

**Complex**	TG3zymogen/Ca^{2+} +Fab	TG3cleaved/Ca^{2+} +Fab	TG3cleaved/Ca^{2+} +Fab +Z-DON	TG3cleaved/no Ca^{2+} +Fab
**Ca^{2+}**	yes	yes	yes	no
**C1C2**	yes	yes	no	yes
**PDB ID**	8OXV	8OXW	8OXX	8OXY
**ESRF beam line**	ID23-2	ID30A-3	ID30B	ID23-1
**Resolution (Å)**	41.8-1.8	50.0-1.7	52.8-2.5	60.4-2.0
	(1.83-1.80)	(1.73-1.70)	(2.60-2.50)	(2.04-2.00)
**Space group**	$P2_1$	$P2_1$	$P2_1$	$P2_1$
**Unit cell**				
**a (Å)**	81.7	81.5	79.3	80.0
**b (Å)**	94.0	93.5	65.0	92.3
**c (Å)**	91.1	90.6	90.9	90.4
**b (°)**	93.5	92.6	96.9	92.3
**$CC_{1/2}$**	98.7 (45.7)	99.5 (35.9)	99.4 (79.0)	99.2 (59.9)
**Completeness (%)**	99.9 (99.9)	98.7 (99.9)	95.6 (98.7)	99.9 (99.8)
**No. of unique reflections**	127,215 (6308)	146,920 (7313)	30,234 (3536)	88,598 (4486)
**Redundancy**	4.5 (3.9)	3.0 (2.9)	3.7 (3.9)	3.7 (3.7)
**$I / \sigma(I)$**	6.7 (0.6)	7.1 (0.7)	8.9 (2.0)	5.5 (0.9)
**R_{meas} (%)**	12.2 (151)	8.3 (144)	13.5 (77.6)	15.1 (123)
**R_{cryst}/R_{free} (%)**	18.5 / 22.9	17.2 / 21.0	15.4 / 22.2	17.6 / 22.6
***r.m.s.d.* bond lengths (Å)**	0.011	0.012	0.0090	0.0098
***r.m.s.d.* bond angles (°)**	1.71	1.73	1.65	1.66
**B-factors ($Å^2$)**				
**Backbone**	36.7	34.7	44.7	34.3
**side chains**	40.0	38.9	49.3	38.8
**Ligand + buffer molecules**	64.2	54.3	65.4	44.2
**Ions**	34.7	35.4	45.3	-
**Ramachandran plot**				
**Favoured (%)**	99.9	100	99.9	99.9
**Outliers (%)**	0.09	0.00	0.11	0.09

Supplementary Table 3. Analysis of metal binding sites using the CheckMyMetal server (Gucwa *et al*, 2023).

	Atomic contacts	Carboxyl side chain	Main chain carbonyl	Other side chain	Water	Valence[1]	gRMSD[2]	Distance (Å) Ca-O
8OXV								
Site 1	O7 octahedral	Asp229 bidentate	Ala222, Asn225, Asn227	Asn225	1 (H-bond to Asp228)	2	13.4	2.37, 2.49, 2.32, 2.41, 2.38, 2.41, 2.45
Site 2	O7 octahedral	Glu444 bidentate, Glu449	Ser416	Asn394	2 (H-bond to Asp396)	2.3	13.2	2.41, 2.42, 2.33, 2.41, 2.46, 2.18, 2.35
Site 3	O6 octahedral	Asp302, Asp304, Asp325	Ser308	Asn306	1	1.8	11.5	2.42, 2.34, 2.36, 2.33, 2.38, 2.50
8OXW								
Site 1	O7 octahedral	Asp229 bidentate	Ala222, Asn225, Asn227	Asn225	1 (H-bond to Asp228)	2	13.9	2.48, 2.43, 2.35, 2.35, 2.36, 2.41, 2.47
Site 2	O7 octahedral	Glu444 bidentate, Glu449	Ser416	Asn394	2 (H-bond to Asp396)	2.2	11.7	2.45, 2.46, 2.31, 2.42, 2.40, 2.15, 2.58
Site 3	O6 octahedral	Asp302, Asp304, Asp325	Ser308	Asn306	1	1.9	11.3	2.42, 2.31, 2.39, 2.29, 2.34, 2.43
8OXX								
Site 1	O7 octahedral	Asp229 bidentate	Ala222, Asn225, Asn227	Asn225	1 (H-bond to Asp228)	2.2	14.5	2.38, 2.43, 2.39, 2.39, 2.38, 2.38, 2.28
Site 2	O7 octahedral	Glu444 bidentate, Glu449	Ser416	Asn394,	2 (H-bond to Asp396)	2.2	12.7	2.40, 2.46, 2.38, 2.40, 2.39, 2.22, 2.32
Site 3	O6 octahedral	Asp302, Asp304, Asp310, Asp325	Ser308	Asn306	0	1.7	16.9	2.44, 2.44, 2.40, 2.42, 2.39, 2.41

[1] Valence: Summation of bond valence values for an ion binding site. Valence accounts for metal-atomic contact distances.

[2] gRMSD: R.M.S. Deviation of observed geometry angles (L-M-L angles) compared to ideal geometry, in degrees.

Supplementary Table 4. Overview of hydrogen bonds between Fab DH63-B02 and TG3 core domain.

Fab DH63-B02	IMGT	Atom	TG3 core domain	Atom	Distance (Å) in 8OXV	Distance (Å) in 8OXW	Distance (Å) in 8OXX	Distance (Å) in 8OXY
Heavy chain CDR1								
Asp31	D36	OD1	G243	N	2.87	2.85	2.91	2.83
		O	G243 via H_2O	O	2.74 / 3.36	2.76 / 3.23	2.83 / 3.35	2.65 / 3.18
		O	R269 via H_2O	NH2	2.74 / 3.22	2.76 / 3.11	2.83 / 3.15	2.65 / 3.14
Heavy chain CDR3								
Leu102	L110	O	R245	NH1	2.92	2.83	via H_2O	via H_2O
		O	N259 via H_2O	ND2	2.86 / 2.74	2.91 / 2.94	2.83 / 2.99	2.85 / 3.11
		N	R245 via H_2O	NH2	2.96 / 3.34	2.92 / 3.22	R245 moved	R245 moved
Gly103	G	O	R269	N	2.85	2.81	2.83	2.69
Ser104	S111.1	OG	N259 via H_2O	ND2	2.57 / 2.74	2.67 / 2.94	2.69 / 2.99	2.51 / 3.11
		OG	N259 via H_2O	O	2.77 / 2.71	2.69 / 2.75	3.62 / 2.73	2.81 / 2.73
		O	R269 via H_2O	NH2	3.19 / 3.18	3.06 / 2.94	no H_2O	no H_2O
Asp105	D112.1	OD2	S266	OG	2.75	2.78	2.77	2.85
		OD1	R269	NH1	2.97	2.98	2.96	2.93
		OD1	R269 via H_2O	NH2	2.40 / 3.18	2.65 / 2.94	no H_2O	no H_2O
		N	S266 via H_2O	O	2.97 / 2.90	2.88 / 2.85	3.00 / 2.82	3.01 / 2.84
		N	P267 via H_2O	O	2.97 / 2.70	2.88 / 2.86	3.00 / 2.66	3.01 / 2.75
		N	S263 via H_2O	OG	2.97 / 3.05	2.88 / 2.97	3.00 / 3.09	3.01 / 2.98
Ser106	S112	OG	K262	O	3.24	3.34	3.38	3.41
		OG	N259 via H_2O	O	2.77 / 2.71	2.65 / 2.75	2.62 / 2.73	2.59 / 2.73
Light chain CDR1								
Asp30	D35	O	K262	NZ	2.94	3.02	3.19	2.79
		OD1	K262 via H_2O	NZ	2.77 / 3.00	Asp moved	Asp moved	3.10
		OD1	K261 via H_2O	NZ	2.54 / 2.83	2.47 / 3.06	2.88 / 2.94	Asp moved
Ser31	S36	O	K261 via H_2O	O	2.79 / 2.82	2.54 / 2.82	2.24 / 2.89	2.68 / 2.78
Asn32	N37	ND2	K262	O	3.06	2.97	3.08	3.09
		O	K262 via H_2O	NZ	2.88 / 2.93	2.99 / 2.89	2.86 / 2.61	2.76 / 2.67
Tyr33	Y38	OH	N259	OD1	2.97	2.93	2.70	2.78
		OH	E255 via H_2O	OE2	2.60 / 2.69	2.59 / 2.83	no H_2O	no H_2O
		OH	K258 via H_2O	NZ	2.60 / 3.04	2.59 / 3.10	no H_2O	no H_2O
Light chain CDR2								
Glu51	E56	OE1	N259 via H_2O	OD1	2.60 / 2.91	2.71 / 2.98	no H_2O	no H_2O
		OE1	E255 via H_2O	OE2	2.60 /2.67	2.71 /2.64	no H_2O	no H_2O
Asp52	D57	OD2	K262	NZ	2.92	2.86	2.81	3.00
		OD2	K258 via H_2O	NZ	2.63 / 3.26	2.52 / 3.13	no H_2O	2.61 / 2.67
Light chain CDR3								
Tyr94	Y107	OH	K262	O	2.96	3.00	3.04	2.99
Showing only H-bonds and electrostatic interactions with favourable angles and distances under 3.3 Å between donor and acceptor in at least one of the structures, and only water mediated interactions involving one H_2O. "N" denotes the main chain nitrogen, "O" denotes the main chain carbonyl. The rest are side chain atoms.								

Supplementary Table 5. Overview of hydrogen bonds between Fab DH63-B02 and TG3 C1C2 domains.

Fab DH63-B02		Atom	TG3 C2 domain	Atom	Distance (Å) in 8OXV	Distance (Å) in 8OXW	Distance (Å) in 8OXX	Distance (Å) in 8OXY
Light chain								
Glu51	E56	OE1	N641 via H_2O	O	3.06 / 3.06	2.80 / 3.37	-	-
		OE2	K643 via H_2O	NZ	-	-	-	2.76 / 2.80
Gln54	Q66	NE2	L639	O	3.07	2.96	-	
		OE1	N641	OD1	-	-	-	3.01 / 3.20
Arg55	R67	O	K643 via H_2O	N	3.09 /3.06	2.97 / 3.02	-	-

Showing only H-bonds and electrostatic interactions with favourable angles and distances under 3.3 Å between donor and acceptor in at least one of the structures, and only water mediated interactions involving one H_2O. “N” denotes the main chain nitrogen, “O” denotes the main chain carbonyl. The rest are side chain atoms.

Supplementary Table 6. Overview of direct hydrogen bonds between residues in the catalytic and C1C2 domains of TG3

Catalytic domain	Atom	C1C2 domains	Atom	Distance (Å) in 8OXV	Distance (Å) in 8OXW	Interactions in 8OXX	Distance (Å) in 8OXY
Interactions with the C1 domain							
Trp237	NE1	Tyr526	OH	2.93	2.83	Z-DON	2.84
Asp246	OD2	Ser566	OG	2.97	2.82	Arg248	2.86
Arg248	NH2	Asp567	OD2	3.13	3.23	Tyr241	3.01
Cys273	N	Tyr526	OH	3.12	3.08	Z-DON	3.25
	SG	Tyr526	OH	2.87	2.91	Z-DON	3.03
Gly317	O	Thr520	OG1	2.66	2.66	-	2.58
Asn318	OD1	Trp522	NE1	2.92	2.87	-	2.88
Ser324	O	Arg571	NH2	Ca^{2+} site 3	Ca^{2+} site 3	Ca^{2+} site 3	2.98
Asn329	N	Tyr526	O	2.96	2.88	Z-DON	3.18
	ND2	Asp589	OD2	3.05	3.26	Z-DON	2.93
Trp410	NE1	Glu583	OE1	3.00	3.06	-	2.93
Asn412	ND2	Glu583	O	2.83	2.64	-	2.62
Interactions with the C2 domain							
Asn177	OD1	Ser635	OG	2.70	2.66	H_2O	2.49
	ND2	Glu633	OE1	2.90	2.83	SO_4^{2-}	2.82
Arg248	O	Lys684	NZ	2.82	2.85	crystal contact	2.81
Asn251	OD1	Lys684	NZ	2.67	2.80	crystal contact	2.73
Glu255	OE1	Lys643	NZ	Lys642 different conf.	Lys642 different conf.	Lys642 different conf.	2.84

Showing only H-bonds and electrostatic interactions with favourable angles and distances under 3.3 Å between donor and acceptor in at least one of the structures. “N” denotes the main chain nitrogen, “O” denotes the main chain carbonyl. The rest are side chain atoms.

References

Ahvazi B, Boeshans KM, Idler W, Baxa U, Steinert PM (2003) Roles of calcium ions in the activation and activity of the transglutaminase 3 enzyme. *J Biol Chem* 278: 23834-23841

Ahvazi B, Kim HC, Kee SH, Nemes Z, Steinert PM (2002) Three-dimensional structure of the human transglutaminase 3 enzyme: binding of calcium ions changes structure for activation. *EMBO J* 21: 2055-2067

Fox BA, Yee VC, Pedersen LC, Le Trong I, Bishop PD, Stenkamp RE, Teller DC (1999) Identification of the calcium binding site and a novel ytterbium site in blood coagulation factor XIII by x-ray crystallography. *J Biol Chem* 274: 4917-4923

Gucwa M, Lenkiewicz J, Zheng H, Cymborowski M, Cooper DR, Murzyn K, Minor W (2023) CMM-An enhanced platform for interactive validation of metal binding sites. *Protein Sci* 32: e4525

Han BG, Cho JW, Cho YD, Jeong KC, Kim SY, Lee BI (2010) Crystal structure of human transglutaminase 2 in complex with adenosine triphosphate. *Int J Biol Macromol* 47: 190-195

Jang TH, Lee DS, Choi K, Jeong EM, Kim IG, Kim YW, Chun JN, Jeon JH, Park HH (2014) Crystal structure of transglutaminase 2 with GTP complex and amino acid sequence evidence of evolution of GTP binding site. *PLoS One* 9: e107005

Liu S, Cerione RA, Clardy J (2002) Structural basis for the guanine nucleotide-binding activity of tissue transglutaminase and its regulation of transamidation activity. *Proc Natl Acad Sci U S A* 99: 2743-2747

Pinkas DM, Strop P, Brunger AT, Khosla C (2007) Transglutaminase 2 undergoes a large conformational change upon activation. *PLoS Biol* 5: e327

Stieler M, Buchold C, Schmitt M, Heine A, Hils M, Pasternack R, Klebe G (2020) Structure-based design of FXIIIa-blockers: Addressing a transient hydrophobic pocket in the active site of FXIIIa. *ChemMedChem* 15: 900-905

Stieler M, Weber J, Hils M, Kolb P, Heine A, Buchold C, Pasternack R, Klebe G (2013) Structure of active coagulation factor XIII triggered by calcium binding: basis for the design of next-generation anticoagulants. *Angew Chem Int Ed Engl* 52: 11930-11934

Yee VC, Pedersen LC, Bishop PD, Stenkamp RE, Teller DC (1995) Structural evidence that the activation peptide is not released upon thrombin cleavage of factor XIII. *Thromb Res* 78: 389-397

Yee VC, Pedersen LC, Le Trong I, Bishop PD, Stenkamp RE, Teller DC (1994) Three-dimensional structure of a transglutaminase: human blood coagulation factor XIII. *Proc Natl Acad Sci U S A* 91: 7296-7300
