# Supplementary material for: Autoantibody binding and unique enzyme-substrate intermediate conformation of human transglutaminase 3
Source: Nat Commun. 2023 Oct 5;14:6216. doi: 10.1038/s41467-023-42004-z (PMC10556103; doi:10.1038/s41467-023-42004-z)
Supplement: Supplementary file 6 — Reporting Summary [file 41467_2023_42004_MOESM6_ESM.pdf]

nature portfolio

Corresponding author(s): Julie Elisabeth Heggelund, Ludvig Magne Sollid

Last updated by author(s): Sep 12, 2023

Reporting Summary

Nature Portfolio wishes to improve the reproducibility of the work that we publish. This form provides structure for consistency and transparency in reporting. For further information on Nature Portfolio policies, see our Editorial Policies and the Editorial Policy Checklist.

Statistics

For all statistical analyses, confirm that the following items are present in the figure legend, table legend, main text, or Methods section.

n/a	Confirmed	
☐	☒	The exact sample size (*n*) for each experimental group/condition, given as a discrete number and unit of measurement
☐	☒	A statement on whether measurements were taken from distinct samples or whether the same sample was measured repeatedly
☒	☐	The statistical test(s) used AND whether they are one- or two-sided<br>*Only common tests should be described solely by name; describe more complex techniques in the Methods section.*
☒	☐	A description of all covariates tested
☒	☐	A description of any assumptions or corrections, such as tests of normality and adjustment for multiple comparisons
☒	☐	A full description of the statistical parameters including central tendency (e.g. means) or other basic estimates (e.g. regression coefficient) AND variation (e.g. standard deviation) or associated estimates of uncertainty (e.g. confidence intervals)
☒	☐	For null hypothesis testing, the test statistic (e.g. *F*, *t*, *r*) with confidence intervals, effect sizes, degrees of freedom and *P* value noted<br>*Give P values as exact values whenever suitable.*
☒	☐	For Bayesian analysis, information on the choice of priors and Markov chain Monte Carlo settings
☒	☐	For hierarchical and complex designs, identification of the appropriate level for tests and full reporting of outcomes
☒	☐	Estimates of effect sizes (e.g. Cohen's *d*, Pearson's *r*), indicating how they were calculated

Our web collection on statistics for biologists contains articles on many of the points above.

Software and code

Policy information about availability of computer code

Data collection	The crystallographic data were collected at the European Synchrotron and Radiation Facility in Grenoble, France.
Data analysis	The crystallographic data were analysed using the CCP4 software suite. Details on software versions have been submitted to the Protein Data Bank (www.rcsb.org) and will be publicly available. 8OXV and 8OXW: Refmac version 5.8.0405, Aimless version 0.7.9, DIALS version 2.2.10, Phaser version 2.8.3, Coot version 0.9.8.7. 8OXX: Refmac version 5.8.0411, Aimless version 0.7.9, XDS version 20220110, Phaser version 2.8.3, Coot version 0.9.8.7. 8OXY: Refmac version 5.8.0405, Aimless version 0.7.9, DIALS version 3.12.1, Phaser version 2.8.3, Coot version 0.9.8.7.

For manuscripts utilizing custom algorithms or software that are central to the research but not yet described in published literature, software must be made available to editors and reviewers. We strongly encourage code deposition in a community repository (e.g. GitHub). See the Nature Portfolio guidelines for submitting code & software for further information.

Data

Policy information about availability of data

All manuscripts must include a data availability statement. This statement should provide the following information, where applicable:

- Accession codes, unique identifiers, or web links for publicly available datasets
- A description of any restrictions on data availability
- For clinical datasets or third party data, please ensure that the statement adheres to our policy

The X-ray model co-ordinates and structure factors have been deposited in the Protein Data Bank (www.rcsb.org) under the accession codes 8OXV [https://doi.org/10.2210/pdb8OXV/pdb], 8OXW [https://doi.org/10.2210/pdb8OXW/pdb], 8OXX [https://doi.org/10.2210/pdb8OXX/pdb] and 8OXY [https://

doi.org/10.2210/pdb8OXY/pdb]. The coordinates will be publicly release upon publication of the manuscript. Raw data of line graphs and ELISA are available in the accompanying Source Data file. Raw data for the MS experiments are available as a separate Supplementary file.

Research involving human participants, their data, or biological material

Policy information about studies with human participants or human data. See also policy information about sex, gender (identity/presentation), and sexual orientation and race, ethnicity and racism.

Reporting on sex and gender	Not relevant, since our study does not involve human participants
Reporting on race, ethnicity, or other socially relevant groupings	Not relevant, since our study does not involve human participants
Population characteristics	Not relevant, since our study does not involve human participants
Recruitment	Not relevant, since our study does not involve human participants
Ethics oversight	Not relevant, since our study does not involve human participants

Note that full information on the approval of the study protocol must also be provided in the manuscript.

Field-specific reporting

Please select the one below that is the best fit for your research. If you are not sure, read the appropriate sections before making your selection.

- [x] Life sciences
- [ ] Behavioural & social sciences
- [ ] Ecological, evolutionary & environmental sciences

For a reference copy of the document with all sections, see nature.com/documents/nr-reporting-summary-flat.pdf

Life sciences study design

All studies must disclose on these points even when the disclosure is negative.

Sample size	Our study reports on structural properties of a single human protein, transglutaminase 3 (TG3). In addition, we describe binding of monoclonal antibodies derived from dermatitis herpetiformis patients to different conformers of TG3. These antibodies have been reported in a previous study (Das et al., doi: https://doi.org/10.1101/2023.05.31.542741), and all antibodies we had available were included in binding studies. One antibody was selected for crystallization as it belongs to a major epitope of DH autoantibodies (epitope group 2) with suggestive evidence that it binds to the catalytic domain of TG3. Sample size calculation is therefore not relevant.
Data exclusions	No data were excluded from the analyses
Replication	All biochemical assays reported in the manuscript were repeated at least once and were successful.
Randomization	Randomization is not relevant to our study, because we did not have individual study groups
Blinding	Blinding is not relevant to our study, because we did not have individual study groups

Reporting for specific materials, systems and methods

We require information from authors about some types of materials, experimental systems and methods used in many studies. Here, indicate whether each material, system or method listed is relevant to your study. If you are not sure if a list item applies to your research, read the appropriate section before selecting a response.

Materials & experimental systems

n/a	Involved in the study	
[ ]	[x]	Antibodies
[ ]	[x]	Eukaryotic cell lines
[x]	[ ]	Palaeontology and archaeology
[x]	[ ]	Animals and other organisms
[x]	[ ]	Clinical data
[x]	[ ]	Dual use research of concern
[x]	[ ]	Plants

Methods

n/a	Involved in the study	
[x]	[ ]	ChIP-seq
[x]	[ ]	Flow cytometry
[x]	[ ]	MRI-based neuroimaging

Antibodies

Antibodies used	For Western blot analysis: Primary antibody: polyclonal goat IgG specific for the C2 domain of TG3 (PA5-37896, Invitrogen).<br>Secondary antibody: Peroxidase-conjugated donkey anti-goat IgG (705-035003, Jackson).<br>For ELISA: alkaline phosphatase-conjugated goat anti-human IgG (2040-04, Southern Biotech).
Validation	Information from vendors:<br><br>PA5-37896 validation: Western blot of Transglutaminase 3 in human tonsil lysate was performed using 35 µg protein in RIPA buffer. Lysates were probed with a Transglutaminase 3 polyclonal antibody (Product # PA5-37896) at a dilution of 0.3 µg/mL and incubated for 1 hour, followed by chemiluminescent detection.<br><br>705-035-003 validation: Based on immunoelectrophoresis and/or ELISA, the antibody reacts with whole molecule goat IgG. It also reacts with the light chains of other goat immunoglobulins. No antibody was detected against non-immunoglobulin serum proteins. The antibody may cross-react with immunoglobulins from other species.<br><br>2040-04 validation: FLISA shows the antibody reacting with human IgG, but not human IgM or IgA. May react with IgG from other species.

Eukaryotic cell lines

Policy information about cell lines and Sex and Gender in Research

Cell line source(s)	Commercial cell line Expi293F, product number A14527, ThermoFisher Scientific
Authentication	Authenticated from the manufacturer: Viability assay >90%, Mycoplasma qPCR assay: negative, Sterility testing: meets specification
Mycoplasma contamination	The cell lines were tested for mycoplasma contamination in the local lab and was confirmed to be negative.
Commonly misidentified lines<br>(See ICLAC register)	No commonly misidentified lines were used.
